# Disaster Medical Assistance Teams After Earthquakes in Iran: Propose a Localized Model

**DOI:** 10.5812/ircmj.8077

**Published:** 2013-09-05

**Authors:** Mohsen Abbasi, M Hossein Salehnia

**Affiliations:** 1 Department of Emergency Medicine, Tehran University of Medical Sciences, Tehran, IR Iran; 2 Department of Emergency Medicine, Isfahan University of Medical Sciences, Isfahan, IR Iran

**Keywords:** Earthquake, Composition, Disaster Medical Assistance Teams, Iran

## Abstract

**Background:**

In the past 10 years, 13 fatal earthquakes have occurred in Iran and led to death of 30,000 people whom most of them were killed in the earlier hours of the disaster. Disaster Medical Assistance Teams are groups of trained medical and non-medical personnel with various combinations that on the optimal conditions are deployed just within 8 hours of notification and are able to work self-sufficiently for at least 72 hours without any outside help and can treat up to 250 patients per day. Currently there are no such rapid-response teams in case of unexpected events in Iran, which causes the responses to such disasters, not to be organized or practiced. For instance, there were many rescue forces in 2003 Bam earthquake but not enough skilled ones to cope with; consequently they themselves became a problem in crisis management instead of solving the problem.

**Objectives:**

In this study, we have investigated which of the following is more efficient: changing the size and combination of the team depending on the type of disaster and environmental conditions or, determine a fixed combination team.

**Materials and Methods:**

Totally, several reasons for dynamic combination and size of the teams are presented. later, earthquake disaster is divided into 3 phases in terms of time including the acute phase (1^st^ to 4^th^ day after disaster), the sub-acute phase (5^th^ to 14^th^day) and the recovery phase (after the 14^th^ day), and finally the appropriate team combinations in every phases are offered.

**Results:**

Regarding to introduction and considering the existing statistics in different legal Iranian resources and by division of the earthquake disaster to three phases including acute phase (1st to the 4th day after disaster), sub-acute phase (5th to 14th day) and recovery phase (after the 14th day)

**Conclusions:**

The countries pioneer in disaster medical assistance teams, now are inclined to deploy different teams consistent with each kind of disasters or with other effective components on the combination of system. Every disaster has its own condition and would require different combination of relief and medical forces. For example, people’s health needs in flood is different from the earthquake

## 1. Introduction

Disaster Medical Assistance Team (DMAT) is a group of trained medical and non-medical volunteers with various capabilities. The major activity of DMATs is clinical treatment in affected areas. The National Disaster Medical System (NDMS) is an interagency program in national level in the USA that provides nationwide medical response to major emergencies and disasters. NDMS is made up of several teams includes DMATs and some other more special teams such as Disaster Mortuary Operational Response Teams (DMORT), International Medical Surgical Response Team (IMSuRT) and National Veterinary Response Team (NVRT) (1). The ACEP (American College of Emergency Physicians) unit of disaster medicine was established in 1988. Its members are encouraged to participate in different levels of research, education, emergency management and participation in DMAT (2). DMATs are small mobile hospitals with members who have various capabilities such as flexibility, innovation, consistency with unknown conditions, stress management, empathy morale, high physical capability and working in hard conditions (3). Disaster specialists classify and triage the patients according to the probability of their survival that it means "to do the most good for the most people" (4). Normally, after separating the people who can walk, rapid assessment of perfusion, respiration and consciousness level has done and patients triage in three groups: red (immediate), yellow (delayed) and black (dead people) (5).During treatment of patients the facilities and time should be managed based on the number of patients, severity of damages, danger in disaster scene, etc. Also, information about regional disease and health condition of the society before deploying the team is helpful.

NDMS has developed a standardized DMAT evaluation process based on the organizational development, training, supplies and equipment and team staffing. Teams categorize to three readiness level: level-one teams: They are in the highest readiness level. They can deploy at the best condition within 6-12 hours of the disaster occurrence and should continue their activity at least 72 hours without receiving any help from outside and should try to give services to up to 250 victims per day (6). Thus, they should be self-sufficient in terms of technical and medical equipment, water, food, shelter, etc. everybody should carry his personal equipment. The weight limitation of equipment is 30kg for hot areas and 40 kg for cold areas per any member.

Level -two teams: teams that require only training, staffing or equipment adjustment to turn into a first degree team. Third degree team: They are elementary teams without the essential facilities, equipment or personnel for a total team, but their personnel can be used as a substitute for the other teams’ position. The combination of the team is completely varied. The team combination includes physicians, nurses, physician assistants, paramedics, pharmacists, pharmacy assistants, mental health specialists, dentists, environmental and laboratory specialists, emergency medical technicians, etc. Technical or nonmedical members may consist of engineers, radio operators, administrators and logistic, security, mechanics, financial affairs experts, computer specialists etc. For each of the required positions in the team, at least 2 - 3 people should be in the list to have adequate personnel available to be substituted when a member may not be able to be present. A full team deployment is expected to be 33 to 35. A typical team size for a deployment may vary, according to the mission assignment and final combination of the team is determined based on special medical needs in the region. For instance, Strike teams, a concept developed during the Atlanta 1996 Summer Olympic Games were 5 - 6 member squads usually made up of medical personnel that have the capability to move quickly to an affected area to provide limited medical treatment (7). The team being sent to New Guinea by Australia after tsunami of 2004 was composed of 58 members (3).

## 2. Objectives

Earthquake, storm and drought were the most fatal natural disasters in the past 50 years in the world. In Iran, earthquake, drought and flood were the most terrible natural disasters in the past 100 years (8). Earthquake is less predictable but resulting mortality rate is the highest immediately after the quake, so that 71% of deaths occur in the early minutes and 80% of deaths occur during the first 6 hours (9). DMATs may not significantly impact acute survival if they arrive more than 48 hours after the earthquake (10). The 2003 Bam earthquake was a disaster because the number of injured people was exceeded the capacity of Bam hospitals and they needed external rescue aids. Unfortunately, DMAT groups were not deployed immediately to the affected area. According to the report of the world Red Crescent Society in 2003 Bam earthquake despite this, Iran Red Crescent Society had been equipped with only 10 sniffer dogs; they succeeded to save the lives of 1000 in the first 24 hours. However 35 search and rescue international teams arrived in Bam 36 hours after the quake and they could save only 25 people from the debris (11). Thus, the most efficient people to help the afflicted people were local rescue forces. After Bam disaster, despite of the presence of different institutions and organizations including Red Crescent Society of Islamic Republic of Iran, unexpected events organization and other organizations even military forces, the lack of many groups such as non-governmental organizations (NGOs) and the volunteer coordinator organizations were revealed. Considering that, the present study is the first that has been carried out in the case discussed in Iran, which investigate only the combination of DMAT team members and on the results, the proposed combination is offered. Researching into other aspects such as the required equipment and deployment of the team needs separate investigations.

## 3. Materials and Methods

This research is launching a method or scientific execution system and we hope the proposing combination of medical emergency teams will be used as a model to constitution such teams in Iran and other countries. It can be said that the method using to propose a combination of team is combinational use of existing information from previous events and disasters in Iran and other countries, the combination of performance teams similar to DMAT teams including CERT (Community Emergency Response Team), MMST teams: (Metropolitan Medical Strike team), DMATs in different countries in the world and localization of the combination based on different cultural, climatic and geographical conditions in Iran.

In addition, relying on the experience of Bam earthquake, the disaster could be divided into three time-based phases including acute phase (1^st^ to the 4^th^ day after disaster), sub-acute phase (5^th^ to 14^th^ day) and recovery phase (after the 14^th^ day) and the afflicted people’s major needs in every phase were considered in order to determine the final team combination. The explanation of duties in every positions and posts which can be defined in the team and methodical analysis of existence or nonexistence of a particular specialization or position in team discussed with the experienced experts of events management by face to face interviews and the documented experiences of other countries. For example, the orthopedist was excluded from the combination of team because in the most orthopedic surgeries operating right in disaster scene they faced to high infection rate and most of surgeries were not emergent. In this study, we have mostly used articles of the 2003 Bam, 1990 Manjil and Roudbar in Iran, and the 2001 Gujarat and 1994 Los Angeles earthquakes. Analyzing these articles data has been led to offer the team combination.

## 4. Results

Regarding to introduction and considering the existing statistics in different legal Iranian resources and by division of the earthquake disaster to three phases including acute phase (1^st^ to the 4^th^ day after disaster), sub-acute phase (5^th^ to 14^th^ day) and recovery phase (after the 14^th^ day), it seems the best combination for deployment to quake-ridden area in post-earthquake days (phases after the earthquake) is the followings:

**The best combination for acute phase of disaster (the 1^st^ to 4^th^ day after disaster):**

39 people including:

### A. Non-Medical Group: Totally 10 People

1. Team commander: 1 person

2. Commander Deputy: 1 person, preferably 1 experienced head nurse (for commandership duty, in cases when the commander is not in the team temporarily or the successor of the command at rest time of commander as shift)

3.Team secretary: 1 person (due to the shortage of force to register the names of patients at the rest time of secretary, physician or one of the nurses do this duty. Other duties of the secretary are done by the secretary himself in work shifts)

4.Communication officer: 1 person

5.The chief of financial and administrative affairs: 1 person

6.Logistic chief: 1 person

7.Guard: 2people

8.Safety officer: 1 person

9.Home base (nondeployed) support position: 1 person

### B. Medical group: Totally 29 people

1.Admit and triage team 

Trained nurse: 2 people

Stretcher bearer: 2people

2.Treatment team

*black zone:

Nurse assistant: 1 person

*red zone:

Physician: 1 person (emergency specialist, Anesthesiologist, general surgeon specialist or trained general practitioner)

Registered Nurse: 2 people

Nurse assistant: 1 person

*yellow zone:

Physician: 1 person (emergency specialist, general surgeon or trained general practitioner)

Registered Nurse: 4 people

Nurse assistant: 2people

*green zone:

Registered Nurse: 2 people

Nurse assistant: 1 person

3.Transport team

Nurse: 1 person

Emergency technician: 2 people 

Driver: 2 people (according to the number of vehicles and existing ambulances)

4.Health team

Psychotherapist or psychologist: 1 person

Environment health expert: 1 person

5.Midwife: 1 person

6.Pharmacology affairs: 2 people (preferably one pharmacist and one pharmacy assistant)

This combination is flexible. For example, sometimes it is necessary that more people work in triage field or some nurses go with air medical transport team. These decisions are taken by the commander of the team and are notified.

**Suitable combination for sub-acute phase (5^th^ to 14^th^ day):**

37 people with emphasis on health and mental health personnel:

A.Non-medical group: 10people including:

1.Team commander: 1person

2.Commander deputy: 1 person

3.Team secretary: 1 person

4.Communication chief: 1 person

5.The chief of financial and administrative affairs:1 person

6.Logistic chief: 1 person

7.Guard: 2people

8.Safety officer: 1 person

9.Home base (non-deployed) support position: 1 person

B.Medical group: Totally 27 people including:

1.Medical treatment group: 11 people including:

*Triage

Registered Nurse: 1 person

Stretcher bearer: 1person

*outpatient visit:

General practitioner or emergency specialist: 1 person

Internal Medicine Physician: 1 person (unnecessary)

Pediatrician: 1 person (unnecessary)

Gynecologist: 1 person (unnecessary)

*The patients need brief hospitalization or injection

Registered Nurse: 2 people

Nurse assistant: 1 person

 *pharmacological affairs: 2 people (preferably one pharmacologist and one pharmacy assistant)

2.Transport group: 4 people including 

Nurse or emergency technician: 2 people

Driver: 2 persons

3.Health group: 5 people including

Health training expert: 2 people

The experts of environmental health or fighting against diseases: 3 people

4.Mental health group: 4 people including:

Psychiatrist: 1 person

Psychologist or psychotherapist: 3 people

5.Physiotherapist: 1 person

6.Midwife: 2 people

**A suitable combination for recovery phase (after the 14^th^ day):**

At this time, the requirements of the affected area is close to the condition of before disaster and according to the conditions of each region, the combination of team is defined.

## 5. Discussion

The countries pioneer in disaster medical assistance teams, now are inclined to deploy different teams consistent with each kind of disasters or with other effective components on the combination of system. Every disaster has its own condition and would require different combination of relief and medical forces. For example, people’s health needs in flood is different from the earthquake. The characteristics of the affected area and its geographical conditions such as mountainous or desert climate, different seasons of the year and issues like that, have effects on determining the combination of the team. Even in various time periods of a disaster, the deployment of the teams would have a different combination. Medical, health and sanitary needs of the afflicted people in the first days of disaster are different from the next days. According to the experience of Bam earthquake, the disaster can be divided to three phases:

1. Acute phase (the first to the third day after the disaster): in this phase, the main requirement of the field has been trauma management and management of dead people and mental support of injured people.

2. Sub-acute phase (from forth day to two weeks after the disaster): at this time, the major medical requirements of people is controlling chronic diseases such as hypertension, diabetes and giving specialized services for gynecology, otolaryngology(ENT), pediatrics, psychological and infection diseases. In addition, pursuing health problems and prevention of contagious diseases are the priority in this phase.

3. Recovery phase :( after 2 weeks of the disaster): the health and medical services to survivors were closed to the condition of services before earthquake (12).

Therefore, the necessity of mentioned specialized requirements would be less in the following days comparatively with early days; so maintaining the previous combination of the team is a sort of wasting costs. In other words, by the changing in needs and the expectations from team, the combination of the team is not fixed. After the Los Angeles earthquake, the maximum of injured people presented in the first 48 hours. Most of their complaint in the first 3 days was minor trauma such as lacerations and orthopedic injuries. From the third day, primary care conditions predominated and the main complaints changed to medical profile for gastrointestinal, gynecological and miscellaneous needs. The condition returned to base line after 11 days (13).

DMAT teams who are deployed to foreign countries for humanitarian aids have different circumstances. Preferably, these teams should be specialized teams and deployment of general teams with low level of specialization should be avoided. On the other hand, deployment of over-specialized teams is not that advantageous. In addition, the conditions of the disaster and the types of requirement and other mentioned criteria should be considered in deployment in order to dispatch a highly efficient and effective team to help the afflicted people. For exemplar, surgical team who sent to Bam by IMSuRT (International Medical/Surgical Response Team) after the 2003 earthquake were including 56 members including one heart surgeon, a pediatrician, a gynecologist, a trauma surgeon, an anesthesiologist, etc. This team visited 727 patients in Bam and performed only 5 surgeries. Other patients who were visited by this team were suffering from general diseases such as anxiety, gynecologic and pediatric diseases (14). Accordingly, we've concluded that for national deployment, definition of general DMAT teams with a separate combination suit to different situations as type of disaster, afflicted population, time of team deployment to the location, not only is practical, but is economical too.

The combination of the team is divided into medical and non-medical (technical) groups. Non-medical positions are as important as the medical positions. The medical group should include the following 4 groups.

a.Therapy group including physician, nurse, midwife and other therapy personnel.

b.Mental health team including psychologist, psychiatrist, etc.

c.Health group including health experts, health training experts, environmental health engineers and veterinarians, etc.

d.Procurement group and registration including documents specialists and medical register, Stretcher bearers, etc.

Depending on how long after a disaster the team arrives to the scene and on the type of disaster, afflicted population and other conditions, the presence extent of each of four listed groups in the team would be various. The presence of different medical and paramedical specialists in the team is a kind of challenging. One of the most essential and effective specialists who can company the team is emergency medicine specialist. The familiarity with all emergencies and their knowledge about trauma, pediatrics and gynecology, familiarity with different kinds of disasters and methods of response, has made their position to a unique one in the team. Emergency medicine specialists can also be the leader of the team. If an emergency medicine specialist is not in the team, his duties can be delegated to a trained general practitioner or a trained general surgeon. However, these people should attend and pass seriously training courses of disaster and the related skill courses.

Orthopedic surgeon can do casting and splinting and other measurements of orthopedic and start the surgeries if the operation room exists. Except for rare cases, orthopedic emergencies are seen rarely in disaster scenes. The rare cases include dislocations and arterial or neural damages. Most of orthopedic emergency measurements are done by an emergency medicine specialist or a trained general practitioner or a general surgeon. There are not good experiences of orthopedic surgeries in disaster field in all over the world. For example, in the 2001 Gujarat earthquake, the trained orthopedists operated in the affected areas by there were high infection after the operation (3). In the 2003 Bam earthquake, most of the orthopedic surgeries were done outside of Bam. About half of 563 hospitalized patients in Tehran were suffering from lower limb, pelvic or upper limb fracture (15). Of 411 admitted patients in Rafsanjani hospital, 60%underwent orthopedic surgeries (16). These surgeries lasted days after the admittance of the patients. Accordingly, it’s better to transfer orthopedic patients outside the disaster field for operation, so the presence of an orthopedist is not necessary in the team. Being accessible in emergency conditions, however, some volunteer orthopedists must necessarily attend training courses. Orthopedic can attend in field hospitals or other equipped centers (e.g. hospitals of the city, when they are not involved in the disaster) and in surgery specialized teams such as IMSuRT.

General surgeon's specialized activities in disaster field include triage, management of burn, neck injuries, vascular damages, airway injuries, acute abdomen and abdominal trauma, difficult urinary catheterization, chest tube, wound management, etc. All the above can be done by an emergency medicine specialist or a trained general practitioner except the procedures needing operation room. Abdominal surgery in disaster scene particularly in the first or second days of the disaster is not logical and decision making for such operation requires some equipment such as ultrasound device or DPL set for diagnosis and operation room for treatment. Management of vascular injuries should be done in the golden time but it is impossible in the disaster field. Totally, a few people from saved ones in the first 24 hours need emergency surgery (17).The presence of a surgeon in the team is not a necessity but specially in case of absence an emergency medicine specialist, the presence of a surgeon is useful. Also, the surgeon should be present in the nearest equipped clinic (e.g. field hospitals being set up around the affected area).

Gynecology& obstetrics are common diseases in disaster. In addition, premature deliveries are possible due to the stress of the event. Menstrual disorders, vaginal infections are exacerbated after the disaster. Unsafe sexual activities and rapes increase after the disaster and family planning is the requirement of any time. All the above actions can be managed by a midwife and a physician (gynecologist, general surgeon, emergency medicine specialist or even general practitioner). Cesarean section is an exception. If a surgeon is in the team, the presence of a gynecologist is not necessary. If a surgeon is not in the team, the women who need surgery can be transported to a field hospital. Also, a gynecologist can be present in specialized DMATs such as specialized DMATs of genecology or UNFPA (United nation Family planning Activity) or in field hospitals. 

Most of the experts believe that the most effective people in crisis management are general practitioners and nurses. The position of a general practitioner in the team is imperative. Most of procedures such as immobilizations, wound care, CPR, etc. can be done by a trained general practitioner. Even some procedures such as chest tube, intubation, etc. can be done by a general practitioner who had taken special courses. Controlling chronic diseases such as hypertension, diabetes mellitus and infectious diseases, etc. can be done by an internal medicine physician. Controlling chronic diseases in acute phase of disaster is not a priority but in sub-acute phase (after the third day), should be considered. Although all of these can be done by a general practitioner or emergency medicine specialist, the presence of an internist can be useful after the acute phase of disaster.

Infectious disease specialists can play an important role in coping with bioterrorism and many man-made disasters. In natural disasters, the presence of an Infectious disease specialist can be helpful for observation, planning and management of infectious and parasite diseases being epidemic after acute phase of the disaster. The diagnosis and treatment of common infectious diseases such as respiratory tract infection and gastroenteritis can be done by general practitioners or emergency medicine specialists. In the structure of general DMATs, volunteer Infectious disease specialists should be registered and attend the training courses but their deployment to the event filed is decided based on the type of the event and the deployment time. There is no logical reason for the presence of neurosurgeons in the team. A neurosurgeon should be in an equipped center with a professional operation room and diagnostic devices. This group can attend field hospitals or other equipped centers (e.g. the city hospital, when it is not involved in the disaster) and specially surgery teams such as IMSuRT.

The presence of a pediatrician in the combination of the deployed team in acute phase is not logical, either. In the following phases as children were 1/3 of the patients, the presence of a pediatrician can be useful, but in the absence of a pediatrician, a general practitioner or emergency medicine specialist can play their role. Registering pediatricians and training them and keeping them in reservation for DMAT teams are sensible in special cases. The presence of anesthesiologists is more vital than other specialists due to their ability in CPR, stabilizing the unstable patients and their knowledge about sedative and analgesic drugs. The presence of anesthesiologists for all the specialized DMATs such IMSuRT is necessary. Generally, DMAT team is not a specialized team. Its duty is mostly the initial resuscitation, triage, helping to transport patients and also treatment of some injuries and diseases. DMAT team should not waste its time on time-consuming procedures on patients or their CPR. Its duty is giving the maximum services to maximum people. Because of this, the presence of specialists in the team when they occupy the general positions is not logical. Regarding the teams being sent to abroad, it is better to send a specialized team with adequate facilities. In addition when we are far from acute phase of disaster, deployment of specialized teams is more logical.

The role of a midwife in the team can be useful in all the phases. The midwives can manage most of gynecology & obstetrics diseases and can work as a substitute for a gynecologist and even they can work as a nurse. About half or all positions of female-nurses in the team can be given to midwives. Menstrual disorders, family planning, pregnant women health care, delivery during disaster, prevention of sexually transmitted diseases, treatment of gynecological inflammations and infections and many other diseases are in specialized field of midwives. If the patients need to visit by a gynecologist, midwives should refer them to the hospitals of the region that their address is given to the commander. In case of the presence of gynecologist in the team, the patients refer to her. It is obvious that this group should participate in special disaster training courses.

A nurse has a key role in the team and can appear as the team commander. Doing triage, treatment, medical records, patients transport, etc. can be done by a trained nurse. The presence of a physiotherapist in the team during acute phase is not efficient. If according to climatic and seasonal conditions and the past history of vaccination in the region, vaccination is required, a person should be sent as a vaccinator with adequate equipment. In the previous investigations, 80-90% of the mortality rate in homeless population in emergency conditions is due to one of the following 5 diseases: malnutrition, measles, acute respiratory diseases, diarrhea and malaria. In disasters, the probabilities to be affected by these diseases are high. Measles is one of the main killers of children under 5 years after all disasters and if the children suffer from malnutrition, this urgency is increased (18). Vaccination against rabies and tetanus also should be considered.

The mental health services should be started from the earliest moments after the event and even before the relief forces arrive, by all the people who are already trained for this case. Regarding all the events we are informed before their occurrence such as flood and hurricane mental health teams should take the required actions before the event and train the people. Specialized teams of mental health should visit the injured people for a long time after the event. After the 2003 Bam earthquake, the initial interventions of mental health was started by local and international teams in the second day after the event and within 8 months, about 72000 people underwent psychological and psychiatric interventions (19). One month after the event, 100% of Bam children (7-11 year) were suffering from behavioral disorders, of which 75% were suffering from neurotic disorders, 10% anti-social and 10% were not separable (20). But mental health group has another important duty and it is mental support of team members. Using narcotics, alcohol, drugs and sexual intercourse to reduce the stress of team members are forbidden. It seems that in a DMAT general team, the presence of a psychiatrist is not necessary in acute phase of disaster. Its duty such as drug prescription can be done by general practitioner or emergency medicine specialist.

Supervising of water and food safety and sanitation, proper disposal of excreta, garbage and wastage; supervising of burial of corpse and obliterating the animal carcasses, combating against insects, rodents and other disease vectors, disinfection of water wells, surface water management, sanitary wastes disposal and supervision of resettlement of homeless are some of sanitary and health unit activities namely environmental sanitation. Health unit has some duties in the team, too. Food health control and their maintenance conditions, disinfection of patient’s discharges and keeping DMAT location and the patient care place clean, disinfection of ambulance and kitchen and supervision of hospital waste disposal are the duties of this unit. Also, we can add a health training expert for teaching health education to afflicted people and non-professional volunteers. The commander of the event can be one of the medical or non-medical members of team but he shouldn’t have another responsibility at the same time in medical or non-medical group. The main crisis is always consisted of many small crises including crisis of water supply network, crisis of electricity and telecommunication network, security and food, etc. Thus, the deployed team to the field should be a self-sufficient team for a practical time period which shows the high importance of non-medical group of the team.
